# Post-marketing signal detection of fruquintinib-associated adverse events using the FAERS database

**DOI:** 10.1097/MD.0000000000048239

**Published:** 2026-04-03

**Authors:** Cui Liu, Hao Zhang

**Affiliations:** a Department of Pharmacy, Chengdu Seventh People’s Hospital (Affiliated Cancer Hospital of Chengdu Medical College), Chengdu, Sichuan, China.

**Keywords:** adverse events, FAERS, fruquintinib, pharmacovigilance

## Abstract

Fruquintinib, a targeted therapeutic agent approved in 2018 for the treatment of metastatic colorectal cancer, is gradually being applied in an expanding range of clinical settings. Given the complexities associated with real-world dosing, a comprehensive evaluation of its safety profile is essential for optimizing clinical decision-making. We conducted a retrospective pharmacovigilance study utilizing a spontaneous reporting system. Reports related to Fruquintinib were extracted through a standardized data cleaning process and cross-validated using 3 complementary signal detection algorithms: proportional reporting ratio, Bayesian confidence propagation neural network, and reporting odds ratio (ROR). This approach was employed to systematically assess the drug’s safety and identify potential adverse event signals. A total of 92 statistically significant safety signals were identified from 1188 independent cases included in the analysis, corresponding to 1836 adverse event reports. The signal distribution exhibited organ system specificity, with gastrointestinal reactions being the most prevalent category. These reactions primarily manifested as typical drug-related toxicities, including diarrhea and nausea. Other notable complications included neurological, respiratory, dermatological, renal, and cardiovascular events. Important aes not mentioned in the instructions, such as bone marrow suppression (ROR = 11.17), peripheral neuropathy (ROR = 4.24) and dehydration (ROR = 3.98), were also discovered. This study systematically characterizes the post-marketing safety profile of Fruquintinib through a multi-algorithm synergistic analysis, confirming that its safety profile aligns with the clinically expected outcomes based on its mechanism of action. Future research should focus on analyzing drug interaction mechanisms and exploring biomarkers to develop a precise risk-benefit assessment model.

## 1. Introduction

Colorectal cancer (CRC), the third most common malignancy globally and the second leading cause of cancer-related deaths, demonstrates substantial global heterogeneity in its disease burden.^[1]^ Epidemiological data indicate significant geographic variations in the global incidence of CRC, with particularly high rates observed in high- and middle-income countries (HICs). These variations are closely associated with factors such as urbanization, dietary changes toward Western patterns, and population aging. Despite improvements in prognosis due to the promotion of healthy lifestyles and increased early screening awareness, 15% to 30% of patients still present with distant metastases at initial diagnosis. The liver is the most common site of metastasis (approximately 50%–70%), followed by the lungs, peritoneum, and lymph nodes. This metastatic pattern is strongly linked to the biological characteristics of CRC and its vascular invasion pathways.^[2]^

Fruquintinib, a highly selective small molecule tyrosine kinase inhibitor targeting vascular endothelial growth factor receptors (VEGFR) 1, 2, and 3, was independently developed by Hutchison Whampoa Pharmaceuticals. It was approved for marketing in September 2018 through the priority review process by the National Medical Products Administration of China. Fruquintinib is indicated for patients with metastatic colorectal cancer (mCRC) who have failed at least 2 prior standard chemotherapy regimens.^[3]^ The drug inhibits vascular endothelial cell proliferation and migration by competitively binding to the intracellular tyrosine kinase domain of VEGFR and specifically blocking ligand-induced phosphorylation of the VEGF receptor. Notably, VEGFR-1 and VEGFR-2 exhibit high expression in colorectal cancer cells, playing a critical role in regulating tumor cell proliferation, survival, and epithelial-mesenchymal transition through activation of the PI3K/Akt and MAPK/ERK signaling pathways. In contrast, the VEGFR-3 signaling axis is primarily involved in regulating lymphangiogenesis.^[4–6]^ Reported adverse events (AEs) include hypertension (38.7%), hand-foot syndrome (27.4%), bleeding (21.9%), abnormal liver function (elevated AST/ALT in 19.3%), proteinuria (15.8%), and hypothyroidism (12.4%). The incidence of grade 3 or higher AEs is approximately 42.6%,^[7]^ which can be managed with dose adjustments and supportive therapy.

The FDA adverse event reporting system (FAERS) is a publicly accessible spontaneous reporting database that includes a vast collection of AEs, medication errors, and product quality complaints submitted by healthcare professionals, manufacturers, and other stakeholders. It offers a valuable source of real-world data for drug safety research, making it an essential tool for identifying potential adverse reactions and assessing the safety of drugs in clinical practice.^[8]^

This study aimed to identify and characterize post-marketing adverse event signals associated with Fruquintinib in the FAERS database from 2023 to 2024 to support clinical risk management and pharmacovigilance practice.

## 2. Methods

### 2.1. Data collection and cleansing

**Figure 1.**
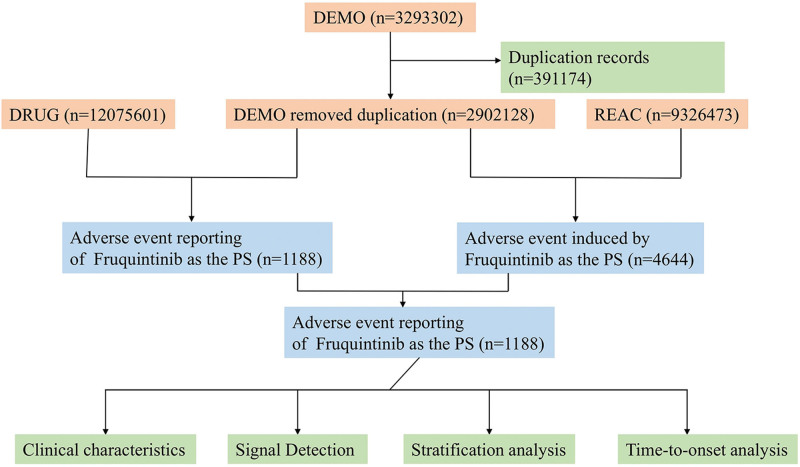
The process of selecting AEs from the FAERS database. AEs = adverse events, FAERS = FDA adverse event reporting system.

The FAERS database includes 7 core data elements: demographic and management information (DEMO), adverse drug reaction data (REAC), patient outcomes (OUTC), drug information (DRUG), treatment start/end dates (THER), reporting source (RPSR), and indications for use/diagnosis (INDI). Adverse drug event (ADE) descriptors were standardized and adapted with reference to the medical dictionary for regulatory activities (MedDRA28.0). Following the FDA’s recommended method for removing duplicate reports, select the PRIMARYID, CASEID, and FDA_DT fields from the DEMO table. Sort the records in the order of CASEID, FDA_DT, and then PRIMARYID. For reports with identical CASEIDs, retain the one with the largest FDA_DT value. Subsequently, for reports with matching CASEID and FDA_DT values, retain the one with the largest PRIMARYID value. ADE reports with the same preferred term (PT) were grouped together and categorized by the system organ class (SOC). A total of 3293,302 patient reports were screened from the FAERS database, with 391,174 duplicates removed. Reports identifying “Fruquintinib” as the primary suspected drug were selected for inclusion in the study from November 2023 to December 2024. As a result, 1188 patients treated with Fruquintinib as primary therapy and 1836 adverse event cases were included for further analysis, as shown in Figure 1.

### 2.2. Signal detection and statistics

Measure of disproportionality (MAD) is a widely used ADR signal detection method both domestically and internationally. In this study, several signal detection methods were used, including reported ratio ratio (ROR), proportional reporting ratio (PRR), and Bayesian belief propagation neural network (BCPNN) as part of the disproportionality analysis method. ROR is one of the classical signal detection methods that help to eliminate bias by providing a more accurate risk estimation. PRR has higher specificity compared to ROR. BCPNN evaluates drugs mainly based on information components and their 95% confidence intervals and has the advantage of integrating and cross-validating data from multiple sources.^[9]^ This comprehensive approach improves the accuracy of safety signal identification, reduces false positives through cross-validation, balances sensitivity and specificity, and enhances the detection of rare AEs by adjusting thresholds and variances. The specific formulas are shown in Tables 1 and 2. Each PT was judgmentally screened according to the thresholds in Table 2, and the judgmental criteria of the 3 algorithms had to be met simultaneously before an ADE signal was generated. The generation of ADE signals suggests a statistical correlation with the drug, and the stronger the signal, the stronger the correlation between the 2. All statistical analyses were performed using R software 4.3.2 and Microsoft Excel for data analysis.^[10]^

**Table 1:** Fourfold table for calculation.

Type of drug	Target AEs	Other AEs	Total
Fruquintinib	a	b	a + b
Non-fruquintinib	c	d	c + d
Total	a + c	b + d	N = a + b + c + d

AEs = adverse events.

**Table 2:** Three major algorithms used for signal detection.

Algorithms	Equation	Criteria
ROR	ROR = ad/b/c	lower limit of 95% CI >1, N ≥3
	95% CI = eln (ROR) ± 1.96 (1/a + 1/b + 1/c + 1/d)^0.5^	
PRR	PRR = a(c + d)/c/(a + b)	PRR ≥2, χ2 ≥4, N ≥3
	χ2 = [(ad-bc)^2^](a + b + c + d)/[(a + b)(c + d)(a + c)(b + d)]	
BCPNN	IC = log2a(a + b + c + d)/(a + c)(a + b)	IC025 >0
	95% CI = E(IC) ± 2V(IC)^0.5^	

BCPNN = Bayesian belief propagation neural network, CI = confidence interval, IC = information component, IC025 = the lower limit of 95% CI of the IC, PRR = proportional reporting ratio, ROR = reporting odds ratio.

## 3. Result

### 3.1. Basic information for adverse event reporting

**Figure 2.**
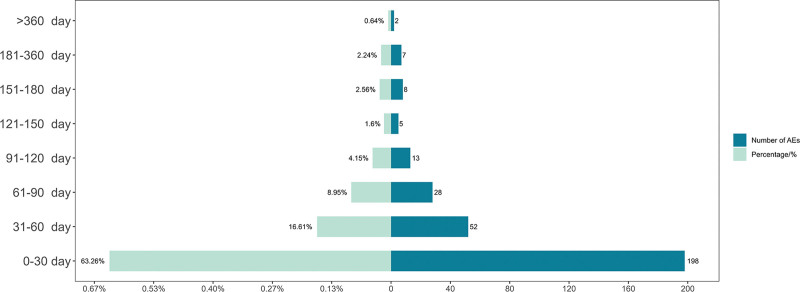
Distribution of AEs over time. AEs = adverse events.

The analysis yielded a total of 1836 AEs related to Fruquintinib, reported between November 2023 and December 2024, involving 1188 patients. The distribution of patients by gender was roughly equal, with a slightly higher proportion of males. The majority of patients (48%) were aged between 18 and 85 years. The United States reported the highest number of adverse reactions, accounting for 848 cases (71.4%), followed by China (15.5%). Detailed information is provided in Table 3. The time to the onset of adverse reactions was defined as the interval between the occurrence of the adverse event and the initiation of Fruquintinib treatment. The most common period for AEs to occur was within the first 30 days of treatment, which accounted for approximately 63% of reported cases (Fig. 2).

**Table 3:** Basic information of fruqunintinib ADE signal.

Basic information	Categorization	Case number, n	Case proportion, %
Gender	Male	614	51.70
	Female	510	42.90
	Unknown	64	5.40
Age	<18	19	1.60
	18–64	323	27.20
	65–85	247	20.80
	>85	11	0.90
	Unknown	588	49.50
Country	China	184	15.50
	United States	848	71.40
	Japan	22	1.90
	France	22	1.90
	Others	112	9.30

ADE = adverse drug event.

### 3.2. Risk signaling SOC

**Figure 3.**
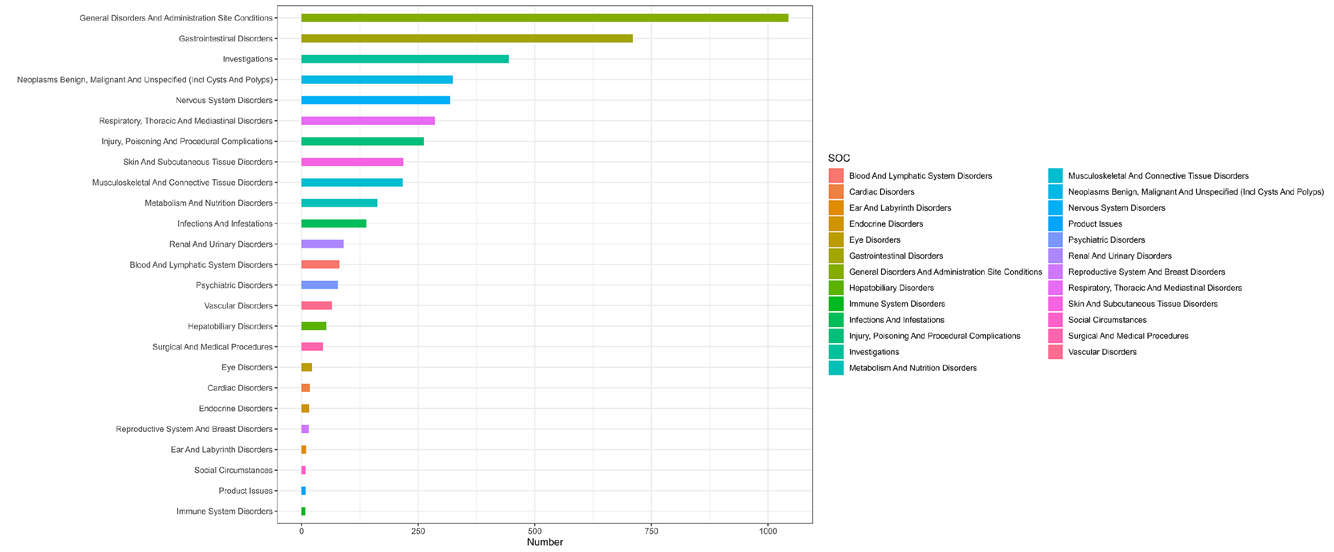
Results of SOC classification of ADE signals. ADE = adverse drug event, SOC = system organ classification.

In this study, a signal detection method was employed to systematically analyze AEs associated with Fruquintinib. The analysis identified 16 SOCs and 92 PTs related to the reported AEs. Based on the frequency of adverse event reports, the top 3 SOC categories were systemic diseases and drug administration site reactions, gastrointestinal disorders, and abnormalities in various laboratory tests. The specific distribution is shown in Figure 3. Quantitative signal detection revealed that the signal intensities of AEs in these 3 SOCs significantly exceeded the preset threshold. Gastrointestinal disorders exhibited the highest signal intensity (ROR = 2.04), followed by laboratory abnormalities (ROR = 1.69), and systemic diseases and site-of-administration reactions, which were statistically significant but had relatively lower signal intensity (ROR = 1.36). The results of this multidimensional analysis may suggest a association between Fruquintinib and these 3 categories of systemic and organ-specific adverse reactions, with gastrointestinal toxicity being particularly prominent.

### 3.3. Risk signaling PT

**Figure 4.**
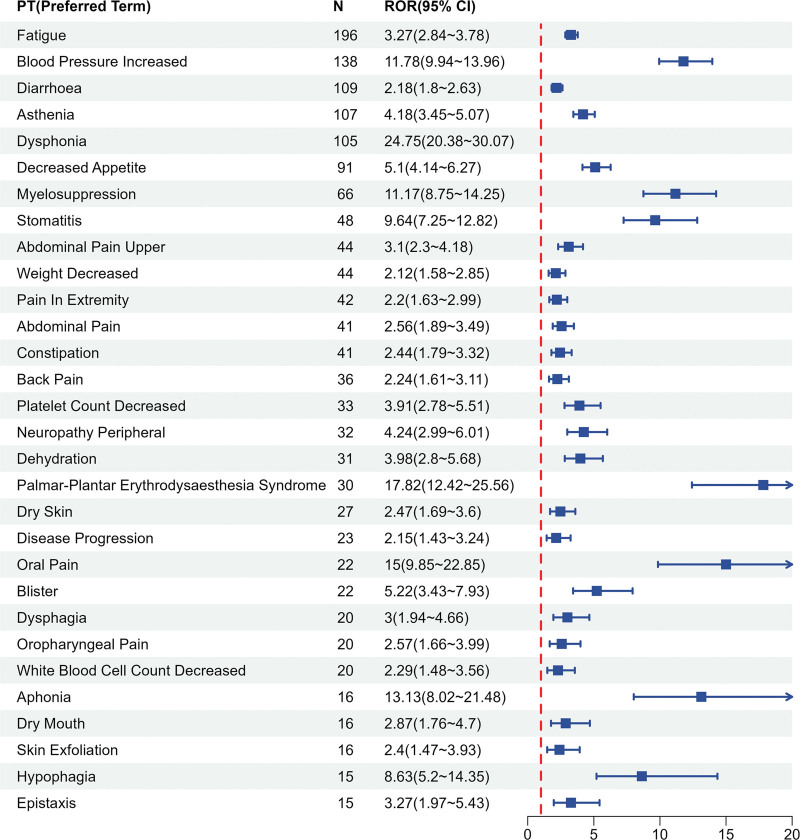
Top 30 PT of frequency. PT = preferred term.

**Figure 5.**
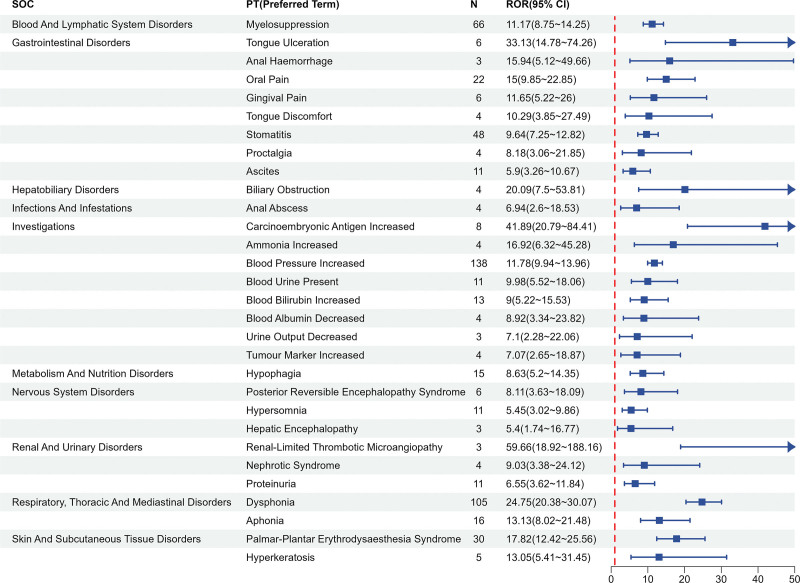
Top 30 PT of signal intensity. PT = preferred term.

In this study, a total of 1836 cases of Fruquintinib-related AEs were systematically evaluated using 3 complementary signal detection algorithms: PRR, Bayesian confidence propagation neural network (BCPNN), and reporting odds ratio (ROR). The analysis was conducted based on data from the FAERS database. After applying strict exclusion criteria, which included nondrug-related events, complications arising from drug administration procedures, pregnancy-specific events, and genetic disorders, 92 statistically significant safety signals were identified. The signal validation results demonstrated a high degree of consistency with the known risk profiles currently listed in the drug’s labeling. Among the 1188 independent patients included in the analysis, the AEs exhibited multisystem involvement. Gastrointestinal disorders, systemic diseases and administration site reactions, and laboratory abnormalities were the most prevalent event categories according to the MedDRA SOC. A frequency analysis of the PTs revealed that fatigue (ROR = 3.27), elevated blood pressure (ROR = 11.78), diarrhea (ROR = 2.18), weakness (ROR = 4.18), and dysphonia (ROR = 24.75) were the 5 most frequently reported AEs (see Fig. 4 for the top 30 rankings). Notably, when ROR values were used to assess signal intensity, the top 5 high-risk signals identified were renal restrictive thrombotic microangiopathy (ROR = 11.17), elevated carcinoembryonic antigen (ROR = 41.89), tongue ulcers (ROR = 33.13), vocal difficulties (ROR = 24.75), and bile duct obstruction (ROR = 20.09). These signals exhibited significantly higher intensities than the traditionally recognized high-incidence AEs (see Fig. 5 for the top 30 signal intensity rankings). Important aes not mentioned in the instructions, such as bone marrow suppression (ROR = 11.17), peripheral neuropathy (ROR = 4.24) and dehydration (ROR = 3.98), were also discovered.

### 3.4. Subgroup analysis

**Figure 6.**
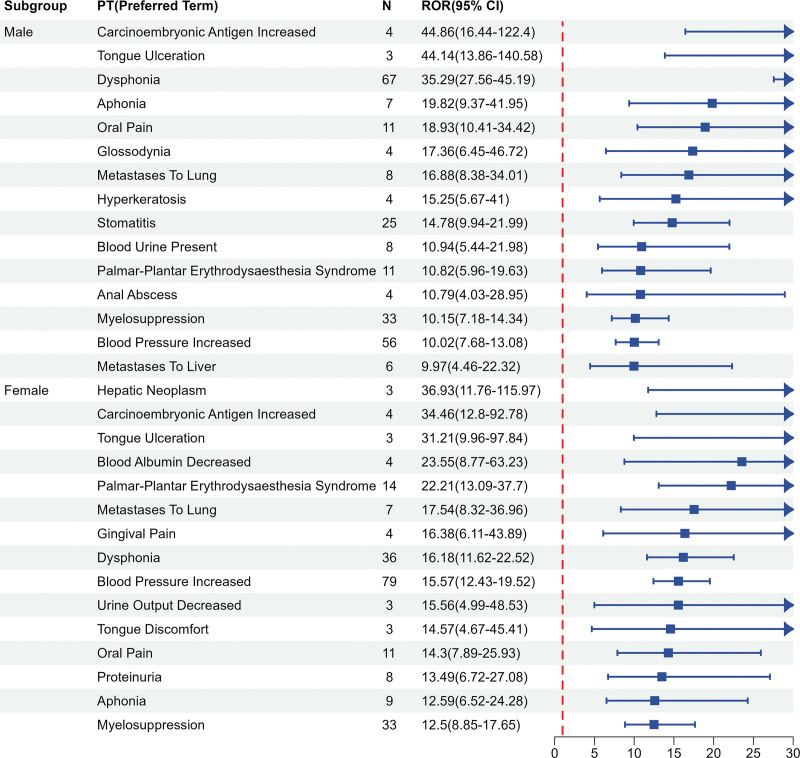
The subgroup analysis in gender.

**Figure 7.**
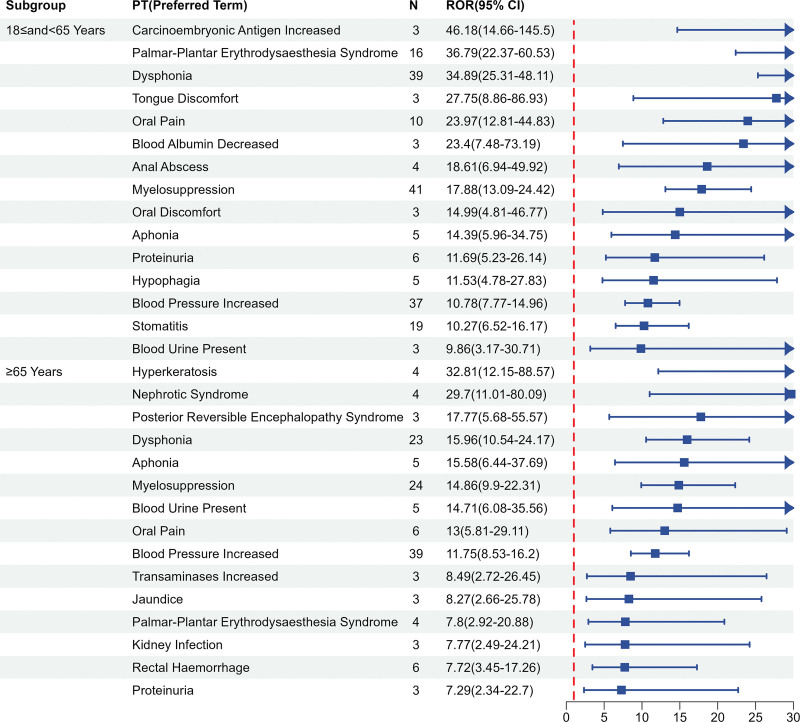
The subgroup analysis in age.

We performed subgroup analyses by gender and age group separately, with a view to exploring whether adverse reactions differed in different populations, but the final results showed a roughly balanced distribution, as shown in Figures 6 and 7.

### 3.5. Comparative study on differences in adverse reactions to VEGFR inhibitors

Conducting an in-depth investigation into the differences in adverse drug reactions (ADRs) among various medications is of paramount importance for refining clinical treatment strategies and safeguarding patient medication safety. With this in mind, we adopted a standardized and rigorous approach to search the FAERS database, focusing specifically on 4 VEGFR inhibitors: Axitinib, Cabozantinib, Sunitinib, and Vandetanib. We systematically gathered reports where these drugs were identified as the primary suspected medications. Subsequently, we applied predetermined screening criteria to select reports that met our study requirements, with the objective of thoroughly examining whether significant differences exist in ADRs among these different VEGFR inhibitors.

The results of our study demonstrated that these VEGFR inhibitors exhibit several common adverse reactions. Specifically, 5 VEGFR inhibitors, including fruquintinib, were found to be associated with the following AEs: palmar-plantar erythrodysaesthesia syndrome, blood pressure increased, blister, decreased appetite, glossodynia, oral pain, stomatitis, dry mouth, hypothyroidism, diarrhoea and proteinuria. The top 5 other adverse reactions are detailed in Table 4. Furthermore, we identified adverse reactions that are specific to fruquintinib, namely bone marrow suppression, peripheral neuropathy, and dehydration.

**Table 4:** Top 5 PT of VEGFR inhibitor.

Drug	PT	N	ROR (95% Cl)
Fruquintinib	Fatigue	196	3.27 (2.84–3.78)
	Blood pressure increased	138	11.78 (9.94–13.96)
	Diarrhoea	109	2.18 (1.8–2.63)
	Asthenia	107	4.18 (3.45–5.07)
	Dysphonia	105	24.75 (20.38–30.07)
Axitinib	Diarrhoea	1835	4.36 (4.16–4.57)
	Fatigue	1571	3.04 (2.89–3.2)
	Hypertension	921	7.27 (6.81–7.77)
	Decreased appetite	772	5.05 (4.7–5.43)
	Dysphonia	768	20.77 (19.33–22.32)
Cabozantinib	Diarrhoea	7798	5.47 (5.34–5.6)
	Fatigue	6262	3.51 (3.42–3.6)
	Nausea	4058	2.36 (2.29–2.44)
	Decreased appetite	3844	7.33 (7.1–7.57)
	Palmar-plantar erythrodysaesthesia syndrome	2585	55.57 (53.29–57.94)
Sunitinib	Diarrhoea	4077	2.84 (2.76–2.93)
	Fatigue	3803	2.16 (2.1–2.24)
	Asthenia	2262	2.68 (2.57–2.8)
	Decreased appetite	2232	4.11 (3.94–4.28)
	Hypertension	1689	3.56 (3.39–3.73)
Vandetanib	Diarrhoea	273	5.1 (4.51–5.76)
	Rash	160	4.2 (3.59–4.91)
	Electrocardiogram qt prolonged	99	33.12 (27.14–40.42)
	Acne	89	12.44 (10.09–15.34)
	Hypertension	70	4.02 (3.18–5.09)

ADE = adverse drug event, CI = confidence interval, PT = preferred terminology, ROR = reporting odds ratio, VEGFR = vascular endothelial growth factor receptors.

## 4. Discussion

The male-to-female ratio among the reported cases was approximately 1:1, with a slight predominance of males. The incidence of the disease was more common among older patients, which aligns with epidemiological findings on the disease.^[2]^ The majority of reports originated from the United States, followed by Europe. This distribution may be influenced by regional variations in drug usage as well as differences in disease incidence across racial groups.^[11]^

Among the 16 SOC categories associated with fruquintinib-related ADE signals, systemic diseases/administration site reactions, gastrointestinal disorders, renal/urinary disorders, and abnormal laboratory test parameters were most frequently reported.^[7]^ This distribution pattern corresponds to the known safety profile outlined in the drug’s prescribing information.

A comprehensive frequency and risk analysis of ADRs associated with fruquintinib revealed that the primary ADRs requiring clinical monitoring include elevated blood pressure, hand-foot skin reaction (HFSR), proteinuria, diarrhea, and oral mucositis. Real-world data from a Chinese cohort (n = 76) further validated these findings. In a single-center retrospective study, the major hematologic toxicities observed were hypertension (9.2%), HFSR (7.9%), and thrombocytopenia (3.9%). Non-hematologic toxicities included oral mucositis (1.3%) and proteinuria (1.3%). Notably, the incidence of ALT elevation (1.3%) and neutropenia (1.3%) in this cohort was lower than that reported in the FRESCO phase III clinical trial, potentially due to differences in baseline hepatic function among the enrolled patients.^[12]^

Shu et al described a case of pemphigus vulgaris (CTCAE grade 3) during fruquintinib monotherapy, which presented with features distinct from typical HFSR: a diffuse maculopapular rash affecting the trunk and proximal extremities, histopathology showing dermal lymphocytic infiltration with eosinophilia, and complete resolution of symptoms after discontinuation of the drug, followed by recurrence upon re-administration.^[13]^ This case may be associated with the need to be vigilant about the potential spectrum of hypersensitivity reactions to fruquintinib. The hypothesized mechanism may involve immune regulation abnormalities due to VEGFR inhibition, although specific signaling pathways remain to be confirmed by in vitro studies.

Zhao et al^[14]^ reported a case of fruquintinib-related renal restrictive thrombotic microangiopathy (RTMA) in a 73-year-old male patient. During treatment for metastatic colorectal cancer with fruquintinib, the patient developed skin peeling of the hands and feet, elevated blood pressure, and increased urine foam. On the 28th day after starting treatment, renal dysfunction prompted referral to the nephrology department, where renal ultrasound and biopsy confirmed RTMA. The kidney injury potentially related to fruquintinib may involve a dual mechanism: direct endothelial cytotoxicity leading to microvascular barrier dysfunction, and compensatory upregulation of pro-angiogenic factors (e.g., Ang-2, FGF-2). This response can disrupt endothelial-podocyte interactions, evidenced by elevated soluble endothelial factors (sVEGFR-1, sEndoglin), which interfere with the localization and function of podocyte proteins (nephrin, podocin) in the glomerular filtration barrier. Guo reported a case of drug-induced glomerular microangiopathy (GMA) potentially related to focal segmental glomerulosclerosis following treatment with tirilizumab and fruquintinib. The patient, who presented with bilateral lower extremity edema and foamy urine, was diagnosed with GMA after renal biopsy.^[15]^

Bleeding risk is another significant adverse effect of fruquintinib. A rare case of cerebellar hemorrhage has been reported in a colorectal cancer patient undergoing treatment with the drug.^[16]^ Studies suggest that VEGF inhibitors contribute to the formation of reactive oxygen species, reduce the activity of the endothelial nitric oxide synthase-nitric oxide (eNOS-NO) pathway, and increase circulating levels of endothelin-1, a potent vasoconstrictor. These effects are important factors influencing blood pressure regulation. Additionally, VEGFR inhibitors compromise vascular integrity and angiogenesis, weaken the vessel wall, impair endothelial cell repair, and disrupt the blood-brain barrier, thereby increasing the risk of hemorrhage.^[17]^ Ledet reported a case of reversible posterior cerebral syndrome in a 41-year-old female patient, who developed acute headache, nausea, and vomiting 2 months after starting fruquintinib. This eventually led to a generalized tonic-clonic seizure, which resolved with symptomatic treatment.^[18]^ This hypothesized mechanism may be related to fruquintinib’s impact on endothelial cell homeostasis, potentially resulting in arterial hypertension and inadequate cerebral perfusion.^[19]^

## 5. Conclusion

These findings potentially related to fruquintinib with side effects that cannot be ignored. In clinical practice, emphasis should be placed on core risk factors such as hypertension, proteinuria, neurological dysfunction, and early renal toxicity in patients, with dynamic monitoring and proactive intervention implemented. Meanwhile, future research needs to delve into the specific mechanisms by which population characteristics, drug dosage gradients, and duration of medication influence the occurrence of adverse reactions.

## 6. Limitations

There are some limitations in this study. The FAERS database is a spontaneous presentation system, and there are problems of omission, misreporting and missing information in the data. The information comes from various sources (e.g., pharmaceutical companies, patients, doctors, etc), and there is a certain degree of reporting bias.^[20]^ Although the deduplication process in this study based on FAERS can mitigate the impact of duplicate submissions, the current data cannot support precise incidence calculations due to underreporting caused by the system’s passivity and the unknowable nature of unreported populations. We sincerely call for further research to explore more robust quantitative methods to overcome the limitations of existing surveillance systems. The dataset timeframe (1 year) limits trend and longitudinal analysis. Although the screening threshold of ADE signal was improved in this study by combining ROR method, BCPNN method and PRR method, the possibility of false-positive ADE signal still could not be ruled out. The generation of ADE signal could only indicate that there is a statistical correlation between the drug and the ADE, while the causal relationship between the drug and the ADE still needs further clinical research and evaluation. The data in this study are mainly from Europe and the United States, and whether they are applicable to the Chinese patient population remains to be verified.

## Acknowledgments

We are grateful to the staff and participants of The FDA adverse event reporting system (FAERS).

## Author contributions

**Writing – original draft:** Cui Liu, Hao Zhang.

**Writing – review & editing:** Cui Liu.
